# Perirhinal input to auditory cortex supports memory-guided sensory perception

**DOI:** 10.1126/sciadv.aed4808

**Published:** 2026-05-22

**Authors:** Luca Godenzini, Ann-Sofie Bjerre, Yi Hu, Lucy M. Palmer

**Affiliations:** ^1^ Florey Institute of Neuroscience and Mental Health, Parkville, Victoria 3000, Australia.; ^2^ School of Medicine, University of Tasmania, Hobart, Tasmania 7000, Australia.; ^3^ Florey Department of Neuroscience and Mental Health, University of Melbourne, Parkville, Victoria 3000, Australia.

## Abstract

Through learning and memory, the cortex dynamically forms sensory associations to guide our behavior in an ever-changing environment. How memory-related brain regions, such as the medial-temporal lobe, influence sensory cortex to support this process remains unclear. Here, we used two-photon calcium imaging of axonal projections from the perirhinal (PRh) cortex to the auditory cortex during learning and generalization of an auditory discrimination task. PRh input in the auditory cortex increased during learning and influenced performance in a categorization task. Chemogenetically silencing PRh input selectively disrupted the activity of auditory cortex layer 2/3 pyramidal neurons, and PRh photoactivation drove behavior toward overgeneralization. These findings highlight PRh input as a key modulatory pathway that shapes cortical dynamics underlying auditory learning and memory-guided sensory perception.

## Teaser

Perirhinal input to auditory cortex flexibly shapes cortical dynamics to support auditory learning and memory-guided perception.

## INTRODUCTION

Learning to associate sensory stimuli with actions and outcomes is a key feature of the brain that enables flexible and adaptive behavior. These sensory associations emerge through dynamic cortical reorganization (*1*–*5*), which is shaped by interactions between cortical and subcortical microcircuits (*5*–*8*). A hallmark of learning is the ability to generalize sensory associations and categorize sensory inputs according to previously learned rules (*9*–*12*). This process likely depends on memory systems to store information about past experiences to guide the retrieval of relevant associations. However, how memory centers of the brain influence sensory processing to support sensory association and categorization remains poorly understood.

Perceptual categorization requires dynamic reorganization and modulation of sensory signaling (*13*), which is thought to depend on top-down inputs delivered to the upper layers of the cortex (*14*–*16*). These inputs target the apical tuft dendrites of pyramidal neurons, which are specialized for actively integrating bottom-up sensory information with top-down contextual signals during sensory-based behavior (*17*–*22*). Known to be modulated by top-down input during learning (*14*, *23*) and important for transforming sensory information into categorical representations (*13*), layer 2/3 (L2/3) pyramidal neurons and their tuft dendrites are ideally positioned to mediate the formation and refinement of sensory associations during learning and categorization (*18*, *24*, *25*).

The perirhinal (PRh) cortex is part of the medial temporal lobe, which forms a major bidirectional communication relay between the cortex and the hippocampus (*26*). Given this unique position between the cortical sensory processing areas and memory centers of the brain, the PRh cortex has long been considered a hub for learning and memory (*27*, *28*). The PRh cortex encodes experience-dependent object features (*29*) and generates a predictive map of learned behavior (*30*). Shown to regulate learning by gating distal apical dendrites in the primary somatosensory cortex (*31*), PRh input shapes cortical computation through targeted modulation of dendritic integration. However, whether and how PRh input contribute to sensory-based learning and categorization remains unknown. Addressing this question is essential for understanding how memory systems interact with sensory circuits to support flexible, memory-guided formation of sensory associations.

## RESULTS

### PRh input in the auditory cortex during learning of an auditory discrimination task

**Fig. 1.**
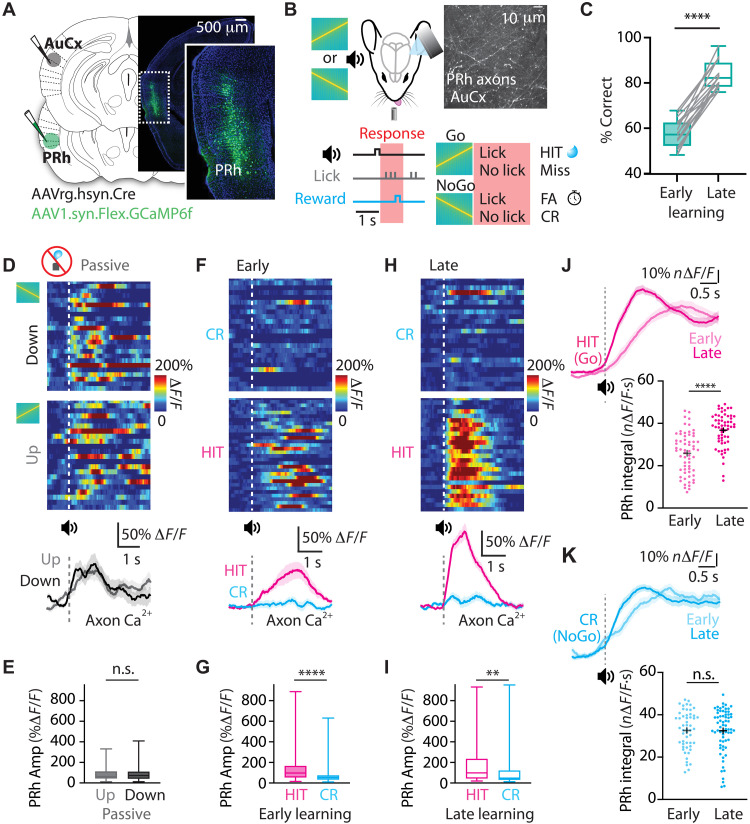
PRh input in the auditory cortex during learning of an auditory discrimination task. (**A**) Schematic of the experimental design. PRh axons projecting to auditory cortex were labeled by injecting the cre-dependent calcium indicator GCaMP6f into the PRh cortex and injecting cre into the auditory cortex. Histological image illustrating Channelrhodopsin-2 (ChR2) expression in the PRh cortex. Inset: Zoom of boxed region. (**B**) Schematic of the auditory discrimination task. Two-photon calcium imaging was performed from PRh axons projecting to the auditory cortex while mice were performing the task. (**C**) Discrimination performance of mice during early and late learning of the auditory discrimination task. Paired *t* test; ****P < 0.0001. (**D**) Top: Heatmaps of calcium activity in a single session and (bottom) average calcium transients in an example PRh axon in the auditory cortex in response to the down sweep and up sweep before learning. (**E**) Average peak amplitude of PRh calcium responses to down and up auditory stimuli before learning. Mann-Whitney test. Amp, amplitude. (**F**) Top: Heatmaps of calcium activity in a single session and (bottom) average calcium transients in an example PRh axon in the auditory cortex during HIT and CR trials in early learning. (**G**) Average peak amplitude of PRh calcium responses during HIT and CR trials in early learning. Mann-Whitney test. (**H**) Top: Heatmaps of calcium activity in a single session and (bottom) average calcium transients in an example PRh axon in the auditory cortex during HIT and CR trials in late learning. (**I**) Average peak amplitude of PRh calcium responses during HIT and CR trials in late learning. Mann-Whitney test. **P < 0.01. (**J**) Top: Overlay of the average normalized activity and (bottom) integral of responsive PRh axons during HIT trials in early and late learning. Mann-Whitney test; ****P < 0.0001. (**K**) Top: Overlay of the average normalized activity and (bottom) integral of responsive PRh axons during CR trials in early and late learning. Mann-Whitney test. n.s., not significant.

Because the PRh cortex is known to influence learning by gating cortical activity (*31*), we first assessed PRh signaling during learning of a sensory-association task. We selectively labeled the PRh-to-auditory cortex pathway by expressing the genetic calcium indicator GCaMP6f in PRh neurons projecting to the auditory cortex (Fig. 1A and fig. S1). Following expression, two-photon microscopy was used to measure calcium signaling in PRh axonal projections within L1 of the auditory cortex. Mice were trained in an auditory Go/NoGo discrimination task where they learned to discriminate the direction of frequency-modulated auditory sweeps (up: 8 to 18 kHz; down: 18 to 8 kHz; 250 ms; 5 octave (oct)/s; Fig. 1B). Following delivery of the “Go” auditory stimulus, mice had to lick a waterspout within a 1-s window (HIT) for a reward (10% sucrose), whereas failing to respond resulted in a time-out (Miss). Following delivery of the “NoGo” auditory stimulus, mice had to withhold their licking response [correct rejection (CR)], while responding led to a time-out [false alarm (FA)]. On average, mice learned this task (>75% correct) after 17.0 ± 1.3 sessions (*n* = 16 mice; Fig. 1C and fig. S2).

To determine how PRh activity changes during learning, we recorded the calcium activity in PRh axonal projections before learning (passive), during early learning (50 to 60% correct), and late learning (>75% correct; expert performance). Classification as “early” and “late” learning was defined for each mouse based on individual learning rates (fig. S2). Before learning, PRh axons responded similarly to up- and down-auditory sweeps (90 ± 9% Δ*F*/*F* versus 89 ± 10% Δ*F*/*F*; *P* = 0.841; *n* = 128 axons, three mice; Fig. 1, D and E) outside of behavioral context. Once training in the task commenced, PRh axons during early learning had different activity patterns in HIT and CR trials (Fig. 1F), with a significant increase in evoked Ca^2+^ responses in HIT trials (155 ± 22% Δ*F*/*F* versus 66 ± 11% Δ*F*/*F*; *P* < 0.0001; *n* = 134 axons, three mice; Fig. 1G). This enhanced activity persisted into late learning (Fig. 1H), with further increases in responses evoked during HIT trials (174 ± 25% Δ*F*/*F* versus 105 ± 17% Δ*F*/*F*; *P* = 0.001; *n* = 109 axons, three mice; Fig. 1I). This increased Ca^2+^ signaling was not primarily due to motor action or reward delivery, as the responses in PRh axons during HIT trials were significantly larger than those evoked by reward delivery alone (88 ± 16% Δ*F*/*F*; *P* = 0.004; *n* = 91 axons, three mice; fig. S3). To test how PRh signaling is refined with learning, we directly compared the PRh axonal responses in early and late learning of the sensory-association task. During HIT trials, PRh axonal activity significantly increased with learning (HIT: 25.8 ± 1.2 *n*Δ*F*/*F*·s versus 36.7 ± 1.0 *n*Δ*F*/*F*·s; *P* < 0.0001; Fig. 1J). This enhanced activity in late learning was not driven by licking, as PRh axonal activity was similar during unrewarded FA behavior (early 27.9 ± 1.3 *n*Δ*F*/*F*·s versus late 27.7 ± 1.7 *n*Δ*F*/*F*·s; *P* = 0.835; fig. S3). In contrast to rewarded HIT behavior, the PRh axonal responses during unrewarded CR trials were similar across learning (32.5 ± 1.2 *n*Δ*F*/*F*·s versus 32.3 ± 1.2 *n*Δ*F*/*F*·s; *P* = 0.922; Fig. 1K), suggesting that the signaling of unrewarded NoGo stimuli is not refined during learning. Together, these findings demonstrate that PRh input to the auditory cortex is dynamically modulated during association learning, with activity selectively enhanced during correct sensory-reward association.

### PRh input modulates neural activity in auditory cortex during an auditory discrimination task

**Fig. 2.**
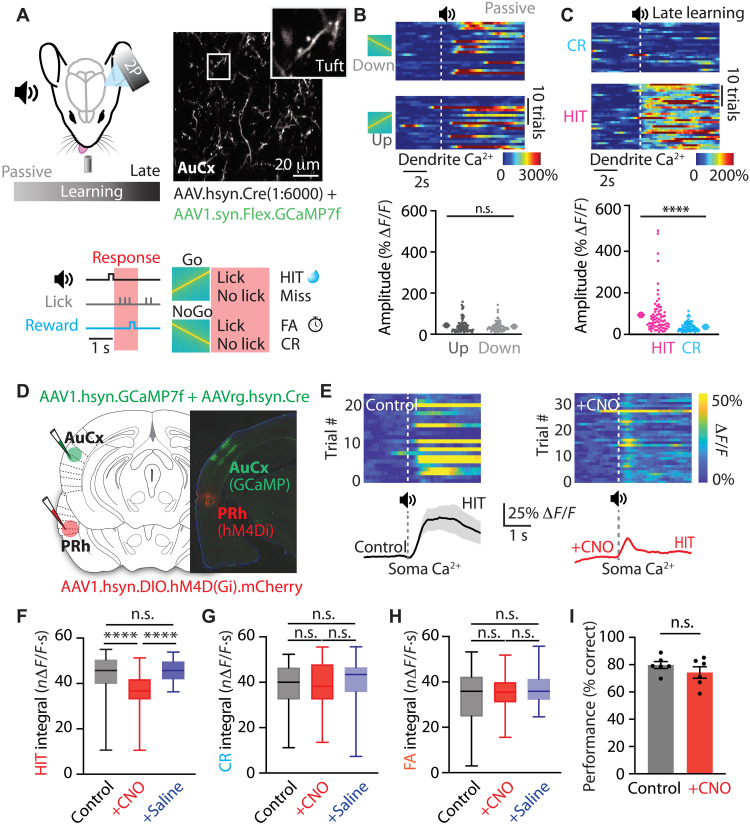
PRh input modulates neural activity in auditory cortex during an auditory discrimination task. (**A**) Schematic of the experimental design. L2/3 pyramidal neurons in the auditory cortex were sparsely labeled with a calcium indicator, and calcium activity in tuft dendrites of L2/3 pyramidal neurons was recorded during learning of the auditory discrimination task. 2P, two-photon. (**B**) Top: Heatmap of calcium activity from an example tuft dendrite in the auditory cortex during one session and (bottom) average amplitude in response to the up and down stimuli before learning. White dashed line: auditory stimulus onset. Mann-Whitney test. (**C**) Top: Heatmap of calcium activity from an example tuft dendrite in the auditory cortex during one session and (bottom) average amplitude of the evoked response during late learning. White dashed line: auditory stimulus onset. Mann-Whitney test; ****P < 0.0001. (**D**) Schematic of experimental design. hM4Di was injected into PRh cortex, and GCaMP was injected into auditory cortex. Histological image illustrating targeted injection sites. L2/3 pyramidal neuron activity in the auditory cortex was recorded during auditory discrimination while silencing PRh input via clozapine N-oxide (CNO) administration. (**E**) Top: Heatmap and (bottom) average calcium activity from example neuron in the auditory cortex during HIT trials in control (black; left) and during CNO (red; right). Dashed line: auditory stimulus onset. (**F**) Integral of somatic calcium responses during HIT trials in control, CNO, and saline. Kruskal-Wallis test with Dunn’s multiple-comparison post hoc test; ****P < 0.0001. (**G**) Integral of somatic calcium responses during CR trials in control, CNO, and saline. Kruskal-Wallis test with Dunn’s multiple-comparison post hoc test; ****P < 0.0001. (**H**) Integral of somatic calcium responses during FA trials in control, CNO, and saline. Kruskal-Wallis test with Dunn’s multiple-comparison post hoc test; ****P < 0.0001. (**I**) Discrimination performance in the task during control and CNO (*n* = 6 sessions from three mice; unpaired *t* test).

To assess how PRh input influences neural activity in auditory cortex, we first measured the activity of pyramidal neurons during the auditory discrimination task. Because feedback projections primarily target apical tuft dendrites, we imaged calcium activity in tuft dendrites of L2/3 pyramidal neurons located within the upper layers of the auditory cortex as mice performed the auditory discrimination task (Fig. 2A). For this, the calcium indicator GCaMP7f was sparsely expressed in L2/3 pyramidal neurons by coinjecting AAV.flex.GCaMP7f.WPE and diluted AAV.hsyn.Cre into the auditory cortex. Similar to PRh axons, dendritic responses to auditory stimuli before (passive) and after learning (no task) were similar across trial types outside behavioral context (0.74 ± 0.10 versus 0.60 ± 0.05 Δ*F*/*F*; *P* = 0.535; *n* = 60 dendrites, six mice; Fig. 2B and fig. S4). Following learning, however, dendritic calcium activity during task performance was significantly greater in HIT trials compared to CR trials (0.93 ± 0.11 versus 0.36 ± 0.02 Δ*F*/*F*; *P* = 0.0001; *n* = 73 dendrites, six mice; Fig. 2C). In contrast, nonlearner mice that failed to learn the task showed no difference in dendritic responses to HIT and CR trials (fig. S4). Somatic calcium activity, measured in a separate subset of experiments, was also modulated by learning (fig. S5). These results indicate that, similar to PRh axons, L2/3 pyramidal neurons in auditory cortex have learning-dependent increases in calcium activity that selectively encode correct sensory-reward association during the auditory discrimination task.

We next asked whether this enhanced cortical activity following learning was driven by PRh input to the auditory cortex. To test this, we selectively expressed the inhibitory DREADD hM4D [AAV.hSyn-DIO-HM4D(Gi)-mCherry] in PRh neurons projecting to the auditory cortex while expressing GCaMP7f in L2/3 pyramidal neurons in auditory cortex (Fig. 2D). In mice expert in the auditory discrimination task, PRh input was chemogentically silenced by clozapine N-oxide (CNO), while the activity of cortical neurons was recorded (Fig. 2E). Chemogenetic silencing PRh input significantly reduced somatic calcium responses during HIT trials compared to control and saline (control: 44.0 ± 0.5 *n*Δ*F*/*F*·s; CNO: 36.8 ± 0.4 *n*Δ*F*/*F*·s; saline: 45.6 ± 0.3 *n*Δ*F*/*F*·s; *P* < 0.0001; Fig. 2F). This effect was specific to HIT trials, as responses during CR trials (control: 38.8 ± 1.1 *n*Δ*F*/*F*·s; CNO: 39.1 ± 1.8 *n*Δ*F*/*F*·s; saline: 40.6 ± 0.9 *n*Δ*F*/*F*·s; *P* = 0.478; Fig. 2G) and FA trials (control: 33.0 ± 1.0 *n*Δ*F*/*F*·s; CNO: 35.4 ± 0.4 *n*Δ*F*/*F*·s; saline: 36.7 ± 0.5 *n*Δ*F*/*F*·s; *P* = 0.206; Fig. 2H) were unaffected by PRh silencing. Notably, PRh silencing diminished the enhanced somatic response during correct rewarded HIT behavior, reducing activity to levels comparable to NoGo and FA trials (*P* = 0.115 and *P* = 0.272; Dunn’s multiple-comparison test). Despite these changes, consistent with previous findings (*31*), chemogenetic suppression of PRh input did not influence behavioral performance in expert mice (control: 1.7 ± 0.1 *d*′; CNO: 1.8 ± 0.2 *d*′; *P* = 0.291; *n* = 6 sessions, three mice; Fig. 2I). Together, these findings illustrate that, similar to PRh axons, neural activity in auditory cortex is selectively enhanced during learned sensory-reward associations, which is largely dependent on PRh input.

### PRh input in the auditory cortex during sensory categorization

**Fig. 3.**
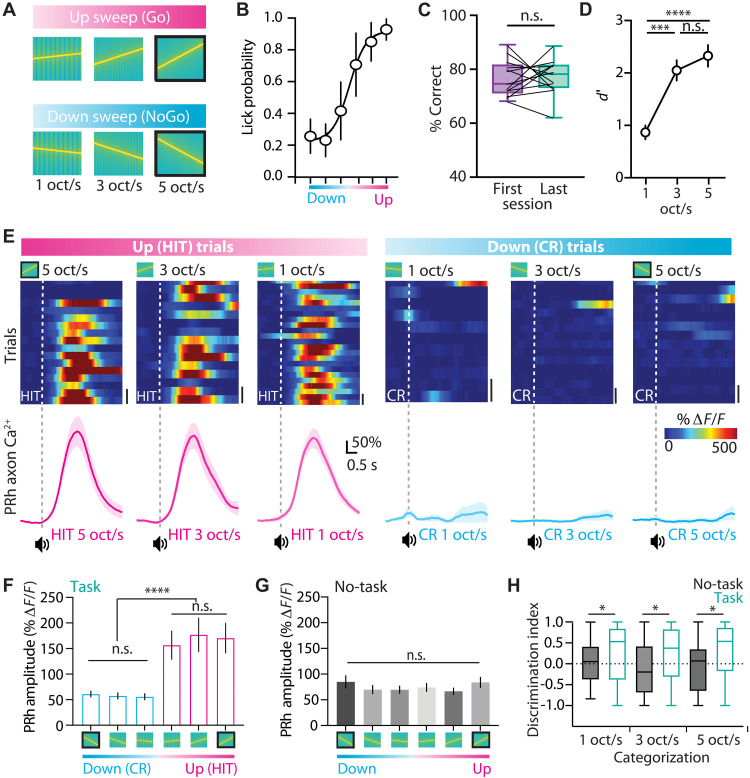
Functional calcium activity in PRh axons in the auditory cortex during auditory perceptual categorization. (**A**) Schematic of the perceptual categorization task. Mice were trained to associate Go auditory stimuli (5 oct/s) with a water reward (black outline). Once expert, they were then presented with novel frequency-modulated sweeps with lower frequency ranges (10 to 16 kHz, 3 oct/s; and 12 to 14 kHz, 1 oct/s). (**B**) Average psychometric curve for performance in the perceptual categorization task. (**C**) Percentage of correct performance in the first (purple) and last (teal) session in the categorization task (*n* = 13 mice, one session each; paired *t* test). (**D**) Discrimination index during the perceptual categorization task for pairs of stimuli (5, 3, and 1 oct/s; *n* = 13 mice, one session each). Kruskal-Wallis test with Dunn’s multiple-comparison post hoc test; ***P < 0.001 and ****P < 0.0001. (**E**) Top: Heatmap and (bottom) average calcium transient for an example PRh axon in the auditory cortex during HIT (left) and CR (right) trials in the perceptual categorization task. (**F**) Peak amplitude of PRh axonal calcium responses during the perceptual categorization task. Kruskal-Wallis test with Dunn’s multiple-comparison post hoc test; ****P < 0.0001. (**G**) Peak amplitude of PRh axonal responses to the auditory sweeps under the passive “no-task” condition (Kruskal-Wallis test). (**H**) Discrimination index of PRh axons for pairs of stimuli comparing task and no-task conditions. Mann-Whitney test; *P < 0.05. Bar graphs represent mean and SEM.

A hallmark of learning is the ability to generalize information and apply learned rules to novel situations. To test whether mice could generalize the rule learned in the auditory discrimination task, we presented novel frequency-modulated sweeps with lower frequency ranges (10 to 16 kHz, 3 oct/s; and 12 to 14 kHz, 1 oct/s; Fig. 3A). In this categorization task, mice were required to lick a reward spout within 1 s of the Go category (up sweep) and withhold licking in response to the NoGo category (down sweep). Unlike subjective categorization tasks, this design used a fixed boundary defined by sweep direction.

Mice successfully generalized the learned rule to the novel stimuli (*n* = 13 mice; Fig. 3B). This generalization was not due to de novo learning, as performance was already high during the first session and remained stable throughout (76.7 ± 1.8% versus 77.2 ± 1.8%; *P* = 0.848; Fig. 3C and fig. S6). As expected, the discrimination index decreased for narrower frequency ranges (5 oct/s: 2.33 ± 0.20 *d*′, 3 oct/s: 2.05 ± 0.19 *d*′, and 1 oct/s: 0.87 ± 0.13 *d*′; Krustal-Wallis test; *P* < 0.0001; *n* = 13 mice; Fig. 3D). These findings indicate that mice can generalize learned behavioral rules to categorize novel frequency-modulated sweeps according to prior experience.

We next examined whether PRh input to auditory cortex similarly generalizes learned information. Calcium activity in PRh axons in L1 was recorded during the categorization task (*n* = 165 axons, three mice; Fig. 3E). PRh axonal responses to novel Go and NoGo stimuli were similar to responses evoked during the learned task (*P* < 0.0001; Kruskal-Wallis test), with no significant difference in response amplitude in either Go trials (5 oct/s: 1.70 ± 0.28 Δ*F*/*F*, 3 oct/s: 1.76 ± 0.32 Δ*F*/*F*, and 1 oct/s: 1.56 ± 0.27 Δ*F*/*F*; *P* > 0.999; Dunn’s test) or NoGo trials (5 oct/s: 0.61 ± 0.05 Δ*F*/*F*, 3 oct/s: 0.57 ± 0.05 Δ*F*/*F*, and 1 oct/s: 0.55 ± 0.05 Δ*F*/*F*; *P* > 0.999; Dunn’s test; Fig. 3F). In contrast, during passive listening (no task), PRh activity did not distinguish sweep direction (*P* = 0.846; Kruskal-Wallis test; Fig. 3G). The discrimination index of PRh axons was consistent across all sweep conditions (5, 3, and 1 oct/s; Fig. 3H), indicating stable encoding of behavioral relevance. Together, these findings suggest that PRh axonal projections in auditory cortex generalize learned sensory associations, maintaining selective signaling for novel but rule-consistent auditory stimuli.

### The influence of auditory cortex on auditory categorization

**Fig. 4.**
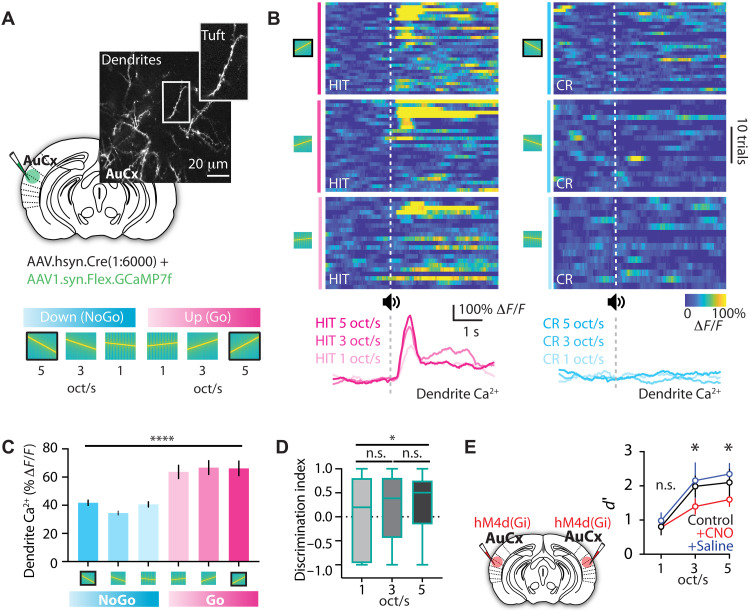
The influence of auditory cortex in the categorization task. (**A**) Schematic of the experimental design. Top: L2/3 of the auditory cortex was sparsely labeled with a mix of cre-dependent GCaMP7f and diluted cre (1:6000). Calcium activity in tuft dendrites of L2/3 pyramidal neurons was recorded using two-photon microscopy during the perceptual categorization task. Bottom: Schematic of the perceptual categorization task. The solid outline highlights the stimuli previously used for training in the auditory discrimination task. (**B**) Activity of an example tuft dendrite in the auditory cortex during the perceptual categorization task. Top: Heatmaps of the calcium response during Go HIT trials (left; magenta) and NoGo CR trials (right; cyan) in 5, 3, and 1 oct/s of trials. Bottom: Trial-averaged calcium activity for the same tuft dendrite in response to 5, 3, and 1 oct/s trials. (**C**) Peak amplitude in tuft dendrites during the perceptual categorization task. Kruskal-Wallis test with Dunn’s multiple-comparison post hoc test; ****P < 0.0001. (**D**) Discrimination index of tuft dendrites in the perceptual categorization task. Kruskal-Wallis test with Dunn’s multiple-comparison post hoc test; *P < 0.05. (**E**) Left: Schematic of the chemogenetic manipulation experiment. Auditory cortex was injected bilaterally with hM4Di. CNO was then applied intraperitoneally to silence activity in the auditory cortex during categorization behavior. Right: Discrimination performance in response to auditory stimuli (1, 3, and 5 oct/s) in the perceptual categorization task during control (black), CNO (red), and saline (blue). Kruskal-Wallis test with Dunn’s multiple-comparison post hoc test; *P < 0.05.

To determine whether L2/3 pyramidal neurons in the auditory cortex also generalize encoding of learned sensory associations, we recorded calcium responses in tuft dendrites of L2/3 pyramidal neurons during the perceptual categorization task (novel frequency-modulated auditory sweeps: 5 oct/s, 3 oct/s, and 1 oct/s; *n* = 609 dendrites, four mice; Fig. 4A). Similar to PRh axons, tuft dendrites had larger calcium responses during Go than NoGo trials (*P* = 0.0001; Kruskal-Wallis test; Fig. 4, B and C). Responses to novel Go and NoGo stimuli were comparable to those evoked during the learned behavior (5 oct/s), with no significant difference across Go trials (5 oct/s: 63 ± 4% Δ*F*/*F*, 3 oct/s: 66 ± 4% Δ*F*/*F*, and 1 oct/s: 66 ± 5% Δ*F*/*F*; *P* > 0.999; Dunn’s test) or NoGo trials (5 oct/s: 40 ± 1% Δ*F*/*F*, 3 oct/s: 34 ± 1% Δ*F*/*F*, and 1 oct/s: 41 ± 1% Δ*F*/*F*; *P* = 0.319; Dunn’s test; Fig. 4C). However, consistent with behavioral performance (Fig. 3D), discrimination responses in tuft dendrites were reduced for narrower frequency sweeps (5 oct/s: 0.30 ± 0.10 versus 1 oct/s: 0.10 ± 0.08; *P* = 0.026; Fig. 4D).

To test whether the auditory cortex is necessary for categorization of learned auditory associations, we chemogenetically silenced it by bilateral injection of the inhibitory DREADD, AAV.hSyn-DIO-HM4D(Gi)-mCherry, during performance in the perceptual categorization task. Silencing the auditory cortex did not alter the number of trials performed (control: 351 ± 24.2 and CNO: 376 ± 12.7; *P* = 0.339; Mann-Whitney Test) but significantly impaired categorization, particularly for broad frequency stimuli with high discrimination (5 oct/s; control: 2.09 ± 0.38, CNO: 1.59 ± 0.19, and saline: 2.34 ± 0.30; *P* = 0.022; 3 oct/s; control: 1.98 ± 0.30, CNO: 1.39 ± 0.22, and saline: 2.15 ± 0.51; *P* = 0.046; Fig. 4E). These findings suggest that dendritic activity in auditory cortex generalizes learned sensory associations and that auditory cortical processing plays an important role in the categorization of learned auditory associations.

### Photoactivation of PRh input in the auditory cortex influences perceptual categorization

**Fig. 5.**
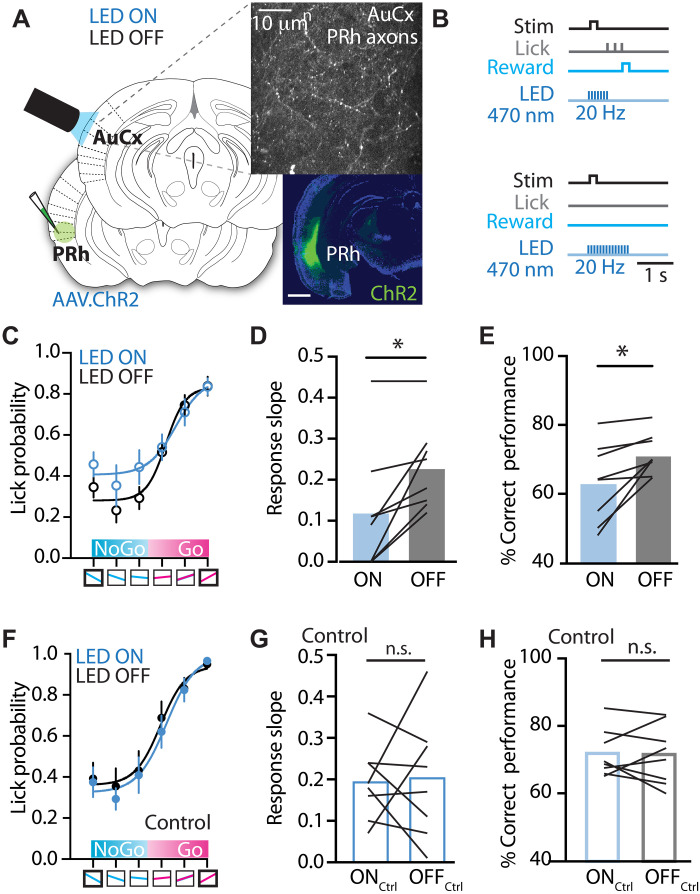
Photoactivation of PRh input during perceptual categorization. (**A**) Schematic of experimental design. ChR2 was injected into the PRh cortex, and a cranial window was implanted over the auditory cortex. Photoactivation of PRh input in the auditory cortex (AuCx) was achieved by delivering blue light from a fiber optic placed over the AuCx. (**B**) Schematic of the trial structure during PRh axonal photoactivation. In LED ON trials, a 470-nm light was delivered at 20 Hz starting from stimulus until first lick detected (top) or, if no lick occurred, until the end of the trial (bottom). (**C**) Performance psychometric curve during the perceptual categorization task in LED ON (blue) and LED OFF (black) trials (interleaved) of an example mouse. (**D**) Slope of the psychometric curves during LED ON and LED OFF trials during PRh photoactivation (*n* = 8 sessions, four mice). Paired *t* test; *P < 0.05. Bar graphs represent mean and SEM. (**E**) Overall performance during the perceptual categorization task in LED ON and LED OFF trials during PRh photoactivation (*n* = 8 sessions, four mice). Paired *t* test; *P < 0.05. (**F**) Performance psychometric curve during the perceptual categorization task in LED ON (blue) and LED OFF (black) trials (interleaved) of an example mouse during control conditions. (**G**) Slope of the psychometric curve during LED ON and LED OFF trials during PRh photoactivation under control condition (*n* = 8 sessions, four mice). Paired *t* test. (**H**) Overall performance during the perceptual categorization task in LED ON and LED OFF trials during control (*n* = 8 sessions, four mice). Paired *t* test; *P < 0.05. Bar graphs represent mean and SEM.

Do PRh axonal projections in auditory cortex directly influence the categorization of learned sensory associations? To address this, the excitatory opsin channelrhodopsin (AAV.ChR2.H314) was expressed in PRh neurons, and their axonal projections in auditory cortex were photoactivated using brief pulses of blue light (10 ms, 470 nm) delivered at 20 Hz via a fiber optic placed over the auditory cortex (LED ON; Fig. 5A). To selectively modulate behavior during the sensory-association task, we delivered light from stimulus onset until the first lick (HIT and FA) or until the end of the response window if no lick occurred (Miss and CR; Fig. 5B). Photoactivation of PRh axons significantly altered behavioral performance, particularly during NoGo trials (Fig. 5C), reducing the slope of the psychometric curve (LED ON: 0.12 ± 0.05 versus LED OFF: 0.23 ± 0.03; *P* = 0.012, four mice; eight sessions; Fig. 5D). Overall, photoactivation of PRh axons reduced task performance, decreasing the percentage of correct behavioral responses (*n* = 8 sessions; *P* = 0.018; Fig. 5E). Control experiments confirmed that these findings were not due to light-emitting diode (LED) exposure alone. Blocking light to the window with a silicon cap eliminated changes in behavioral responses (Fig. 5F), slope of the psychometric curve (LED ON: 0.19 ± 0.03 versus LED OFF: 0.20 ± 0.05; *P* = 0.860, four mice; eight sessions; Fig. 5G), and correct performance (71.9 ± 2.4% versus 71.5 ± 3.1%; *n* = 8 sessions; *P* = 0.861; Fig. 5H). Together, these results demonstrate that PRh input to auditory cortex can bias sensory categorization, driving overgeneralization of learned behavioral responses and leading to an overall impairment in perceptual categorization. The specific effect on NoGo trials suggests that PRh activity may particularly influence active generalized behavior.

## DISCUSSION

Learning requires the formation of sensory associations to support flexible adaption to an ever-changing environment. Changes in neural signaling underlying sensory association need to be generalized so that small changes to the sensory input do not have to rely on relearning. Here, we examined the functional role of PRh axonal input and L2/3 pyramidal neurons within the auditory cortex during auditory discrimination learning and perceptual generalization. By recording calcium activity during learning in an auditory discrimination task, our findings illustrate that PRh axons were selectively active during correct (HIT trials), as animals learned the sensory association. This learning-related PRh activity was mirrored in the tuft dendrites of L2/3 pyramidal neurons, which specifically increased during expert performance (late learning). This increase in amplitude of the dendritic calcium response may reflect burst firing, as previously described (*31*–*33*), although direct voltage recordings are required to test this. Furthermore, the origin of the dendritic calcium signals may be either global (backpropagating action potentials or dendritic calcium spikes) or local (*N*-methyl-d-aspartate spikes) (*34*), which remains an interesting avenue for future studies.

Overall, our findings suggest that learning signals emerge in L2/3 pyramidal neurons following learning, which, as previously described (*31*), may be driven by input from the PRh cortex onto their tuft dendrites. Chemogenetic inactivation of PRh input selectively abolished learning-related enhanced activity in L2/3 pyramidal neurons during correct sensory-reward association, illustrating a causal role for PRh input in shaping cortical learning signals. Together, these results illustrate that top-down PRh input drives dendritic modulation in L2/3 pyramidal neurons during learning and highlights the role of this pathway during memory-guided sensory perception.

The PRh cortex is known to be involved in learning and abstract rule encoding (*30*, *31*). Consistent with this, our findings illustrate that PRh axonal inputs to the auditory cortex were strongly active during task performance, but not during passive stimuli and reward delivery alone, and were remodeled throughout learning. These results confirm that PRh signaling is not primarily related to arousal or thirst, as, despite equivalent arousal and reward, calcium responses in PRh axons differed significantly between early and late learning. In addition, PRh input activation to reward delivery alone do not match the robust responses observed during HIT trials, indicating that PRh signaling is learning and task related rather than driven by general motivational states. Photoactivation of PRh inputs during perceptual categorization led to behavioral overgeneralization. This is highlighted by the stronger effect observed when activating PRh input during NoGo trials compared to Go trials. PRh signaling is already high in response to all Go stimuli in the categorization task (1, 3, and 5 oct/s), and photoactivating this pathway during Go stimuli would not provide additional PRh input to further drive the behavior. Silencing PRh input altered cortical activity without changing behavioral performance; however, this is likely because the PRh cortex was only unilaterally manipulated, which may not be sufficient to affect behavior. Together, these results highlight that PRh-cortical inputs encode task rules (*30*), which are refined during learning and highlight the functional importance of PRh inputs in gating dendritic signaling in sensory cortex to support association learning and generalization during perceptual categorization.

PRh cortex targets many brain regions aside from the auditory cortex, including entorhinal cortex, prefrontal cortex, other sensory cortices, amygdala, and striatum. Collectively, these projections position PRh as a hub linking sensory representations with memory- and emotional-based circuitry. In addition to PRh cortex, the auditory cortex is the target of top-down input from other brain regions including the thalamus (*35*, *36*), prefrontal cortex (*37*, *38*), secondary motor cortex (*39*, *40*), and posterior parietal cortex (*41*). These inputs to auditory cortex may, along with PRh inputs, contribute to shaping dendritic activity during learning.

Generalization and categorization of learned behavior are crucial for maintaining stable behavior despite variations in sensory information, and it may also serve to optimize brain efficiency (*42*). Our findings illustrate that this process involved the interaction of memory systems (PRh cortex) and sensory cortex. We found that both PRh axonal input and tuft dendritic activity were generalized according to the learned category, indicating that this pathway supports perceptual categorization. By integrating top-down task-related inputs with bottom-up sensory information (*43*), cortical dendrites can provide a built-in associative mechanism to support generalization during learning. Categorization of learned information occurs in various cortical regions including posterior parietal (*41*, *44*), auditory (*13*, *45*), somatosensory (*46*), olfactory (*47*), prefrontal (*10*, *48*–*52*), and visual (*53*–*55*), as well as in the hippocampus (*56*, *57*) and basal ganglia (*58*–*61*). Because it is widespread throughout many different brain structures, cellular encoding of generalization might be a general principle involved in the processing of learned information. In summary, our findings reveal that PRh inputs gate dendritic activity in the auditory cortex supporting both learning and generalization of sensory association, providing a general circuit mechanism for memory-guided sensory perception.

## MATERIALS AND METHODS

All experiments were conducted in strict accordance with the Code of Practice for the Care and Use of Animals for Scientific Purposes (National Health and Medical Research Council, Australia) and guidelines given by the veterinary office at the Florey Institute of Neuroscience and Mental Health.

### Surgical procedures

Mice [C57BL/6; postnatal day 30 (P30) to P55; females] were anesthetized with isoflurane [1 to 3% in O_2_ (0.75 liters/min)], and body temperature was maintained at 36° to 37°C. Eye ointment was applied to prevent dehydration, and meloxicam (1 to 3 mg/kg; Ilium) was intraperitoneally injected for anti-inflammatory action. The skin was disinfected with ethanol 70% and betadine, before a small slit was made to expose the skull. A small craniotomy (0.5 × 0.5 mm) was then made over the region of interest (ROI), while the dura was left intact.

For sparsely labeling the auditory cortex with a Ca^2+^ indicator, a mix of Cre-dependent genetic Ca^2+^ indicator GCaMP7f [AAV1. Syn.Flex.GCaMP7f.WPRE.SV40; 7 × 10^12^ vector genomes (vg)/ml] and diluted Cre (1:60040; AAV1.hSyn.Cre.WPRE.hGH; original titer: 7 × 10^12^ vg/ml) was injected into L2/3 (at a depth of 450 μm) at the stereotaxic coordinates of −2.5 mm from bregma and 4.5 mm from midline. Layer specificity of the injection site and identification of the recorded cell-type were confirmed after each experiment by visualizing the entire dendritic arbor and location of the associated somata. For axonal imaging, we injected a cre-dependent Ca^2+^ indicator (AAV1.Syn.Flex.GCaMP6f.WPRE.SV40; 7 × 10^12^ vg/ml) in the PRh cortex (−1.8 mm from bregma and 4.1 mm from midline and −4.2 dorsoventral respect to bregma; 60 nl) and a retrograde cre (AAVrg.hSyn.Cre.WPRE.hGH; 100 nl; 7 × 10^12^ vg/ml) in the auditory cortex (coordinates as above). For axonal photoactivation, we injected Channelrhodopsin-2 [ChR2; AAV1.hSyn.ChR2(H134R)-eYFP.WPRE.hGH; 7 × 10^12^ vg/ml] in the PRh cortex (same as above;100 nl). For chemogenetic manipulation, we injected the inhibitory DREADD hM4D [pAAV1-hSyn-hM4D(Gi)-mCherry; 7 × 10^12^ vg/ml] in the auditory cortex (same as above; 150 nl in each emisphere). For silencing PRh axons while recording auditory cortex L2/3 pyramidal neuron activity, we injected the genetic Ca^2+^ indicator GCaMP7f (AAV1.Syn.GCaMP7f.WPRE.SV40; 100 nl; original titer: 3.5 × 10^12^ vg/ml) into the auditory cortex (coordinates as above) together with a retrograde cre (AAVrg.hSyn.Cre.WPRE.hGH; 100 nl; 3.5 × 10^12^ vg/ml) while injecting a cre-dependent inhibitory DREADD hM4D [pAAV1-hSyn-DIO-hM4D(Gi)-mCherry; 7 × 10^12^ vg/ml] in the PRh cortex (same as above; 60 nl).

To implant a headpost and a cranial window, the connective tissue of the scalp was removed, and a craniotomy was performed (3-mm diameter) over the auditory cortex using a disposable biopsy punch (3 mm). A double window, created by putting together two coverslip of different sizes (3 to 3.5 mm) using ultraviolet curable glue, was placed over the craniotomy and sealed with glue and dental cement. A custom-made metal headbar was then attached to the skull using dental cement (C&B Metabond, Parkell). Mice were returned to their home cage for at least 3 days before habituation and behavioral training commenced.

### Auditory discrimination and categorization task

After recovery from the surgical procedures, mice were gradually habituated to head fixation and the recording setup for 4 to 9 days. Once habituated, mice were water restricted (1 ml/day on restriction days; ad libitum access to food), and behavioral training commenced. Here, auditory stimuli were 250-ms sound sweeps linearly increasing (8 to 18 kHz) or decreasing (18 to 8 kHz) in frequency. We used a range of stimulus frequencies, which covered the peak of the mouse hearing range and would reliably recruit activity within the auditory cortex. Auditory stimuli were delivered through an electrostatic speaker (ES1, Tucker Davis Technology) placed ~5 to 10 cm away from the contralateral ear at a sound pressure level of ~65 dB. Initially, mice were exposed to a Go auditory stimulus paired with an automatically delivered sucrose reward (10 μl; 10% sucrose in water). Once mice consistently retrieved the sucrose water from the spout, training in the Go-NoGo auditory discrimination task commenced. In this stage of the task, reward was only dispensed if the mouse licked the reward spout within a 1-s window following the delivery of the auditory Go stimulus. Conversely, mice were required to refrain from licking after NoGo stimuli. The intertrial interval was randomized between 1 to 8 s, with a new trial initiating only when the mouse refrained from spontaneous licking for at least 1 to 2 s. NoGo trials were initially introduced at a 20:80 ratio compared to Go trials and gradually increased to a 50:50 ratio. If mice exhibited a high rate of FAs (licking after the NoGo auditory stimulus) or Miss (no lick after the Go auditory stimulus), a time-out punishment (1 to 6 s) was implemented. Once mice achieved an expert level (>75% correct response for at least three consecutive days), they progressed to the categorization task. In this task, the structure remained the same, but additional auditory stimuli of shorter frequency ranges (10 to 16 or 12 to 14 kHz) were presented alongside the trained auditory stimuli (8 to 18 kHz) with the same probability. Mice received a sucrose reward for correctly licking after a sound sweep increasing in frequency (8 to 18, 10 to 16, and 12 to 14 kHz) and avoided a time-out by refraining from licking in response to sound sweeps decreasing in frequency (18 to 8, 16 to 10, and 14 to 12 kHz). Mice normally performed around 300 to 400 trials/day per session. During imaging in the discrimination task, sessions were split into blocks of 60 to 80 trials to allow recording more than one field of view on each day. Custom-written behavioral training, testing protocols, and auditory stimuli were implemented on MATLAB (MathWorks) within the Bpod (Sanworks) platform.

### Two-photon calcium imaging

Two-photon imaging was performed through the cranial window implanted in mice previously transfected with the Ca^2+^ indicator. In either early or late learning conditions, Ca^2+^ transients were recorded from tuft dendrites, soma, or axons within the auditory cortex. Axons and dendrites were recorded within L1 of the auditory cortex, between 10 to 100 μm from the surface of the brain. Soma of L2/3 pyramidal neurons was recorded at a depth of 150 to 250 μm from the surface of the brain. The genetically encoded Ca^2+^ sensor (GCaMP6f or GCaMP7f) was excited at 920 nm (~30 mW at the back aperture) with a titanium sapphire laser (140-fs pulse width; Spectra-Physics Mai Tai DeepSee) and imaged on a Sutter MOM through a 16× Nikon objective (0.8 numerical aperture). Emitted light was passed through a dichroic filter (565dcxr, Chroma Technology) and short-pass filtered (ET525/70-2p, Chroma Technology) before being detected by a GaAsP photomultiplier tube (Hamamatsu). Images were acquired at a frequency of 30 Hz (512 × 512 pixels) using the ScanImage software (Vidrio Technologies). All imaging from tuft dendrites was performed in higher-order branches beyond the bifurcation point. All recorded dendrites were traced down to the soma, and only those dendrites confirmed as extending from somata located in L2/3 were included in the analysis. Because of the curvature of the skull in the auditory cortex, tracking the same population of axons or dendrites over long period of time (>2 weeks) was not possible.

### Calcium imaging data analysis

All sessions with motion in the *z* axis were manually excluded from the analysis. The recordings that passed the quality test were motion corrected, and ROI were automatically detected with suite2P. Raw fluorescent data were exported into MATLAB (MathWorks) and analyzed with custom-written scripts. Ca^2+^ responses were smoothed using a Savitzky-Golay filter with a second-order polynomial and a seven-sample window. Activity across all trials was averaged, and ROIs were considered active if the integral value of the average response detected in a 2-s window from stimulus onset crossed a threshold value of 3 SD measured on the baseline (2 s before stimulus onset). Peak amplitudes were measured on active ROIs using the “findpeak” function in MATLAB. In some cases, activity for each ROI was normalized to the maximum value across all trials. To compute the discrimination index, we used the mean fluorescent value measured in a window of 1 s from stimulus onset in responsive ROIs. The discrimination index was then calculated as (stim*_a_* –stim*_b_*)/(stim*_a_ +* stim*_b_*) for pairs of stimuli used in the categorization task (5, 3, and 1 oct/s).

### Photoactivation of PRh axons

Mice were injected with ChR2 [AAV1.hSyn.ChR2(H134R)-eYFP.WPRE.hGH] in the PRh cortex, and a cranial window was placed over the auditory cortex. Mice were trained in the discrimination task, and, after they reached expert performance, they were tested in the categorization task. Photoactivation of PRh axons was achieved by delivering train of light pulses (470 nm, 10 ms, 20 Hz; ~10 to 15 mW outside of the brain) through an optical fiber (M89L01, Thorlabs) directly placed above the cranial window in the auditory cortex. A LED driver (LEDD1B, Thorlabs) coupled to a 470-nm LED (M470F3, Thorlabs) and triggered by an Arduino (with a custom-made script) was used to generate the train of light pulses (20 Hz). Trials with the train of light pulses (LED ON) and without (LED OFF) were randomly interleaved at a rate of 50% each. To reduce confounding effect from the contralateral side, the ear contralateral to the speaker (and photostimulation side) was blocked with a piece of cotton. In control sessions, a silicon cover (Kwik-Cast, WPI) was placed over the window to block light access to the brain. The behavioral protocol was developed and delivered on MATLAB through the Bpod platform. Psychometric curves were fitted using the FitPsyche package for MATLAB, and behavioral data were further analyzed with custom-written scripts on MATLAB.

### Statistics and data analysis

Data were tested for normality, and parametric or nonparametric test were used accordingly using the software GraphPad Prism (version 9.0.0). Normally, for paired data, Wilcoxon matched-pairs signed-rank test was used for statistical analysis, and, for unpaired data, Mann-Whitney test was used for statistical analysis. Statistical tests are reported in each figure caption. All data are reported as mean value with SEM.
