# Supplementary material for: Perirhinal input to auditory cortex supports memory-guided sensory perception
Source: Sci Adv. 2026 May 22;12(21):eaed4808. doi: 10.1126/sciadv.aed4808 (PMC13196781; doi:10.1126/sciadv.aed4808)
Supplement: Supplementary file 1 — Figs. S1 to S6 [file sciadv.aed4808_sm.pdf]

Science Advances
AAAS

Supplementary Materials for

Perirhinal input to auditory cortex supports memory-guided sensory perception

Luca Godenzini *et al.*

Corresponding author: Luca Godenzini, luca.godenzini@hifo.uzh.ch; Lucy M. Palmer, lucy.palmer@florey.edu.au

Sci. Adv. **12**, eaed4808 (2026)
DOI: 10.1126/sciadv.aed4808

This PDF file includes:

Figs. S1 to S6

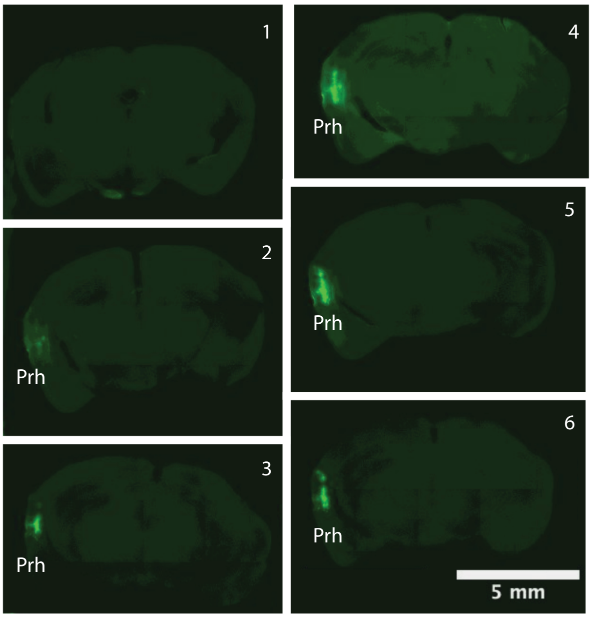

Fig. S1. Additional histological validation of PRh targeting. Series of sections (thickness = 100 μm) from an example mouse showing localised and targeted expression of GCaMP6f in PRh cortex.

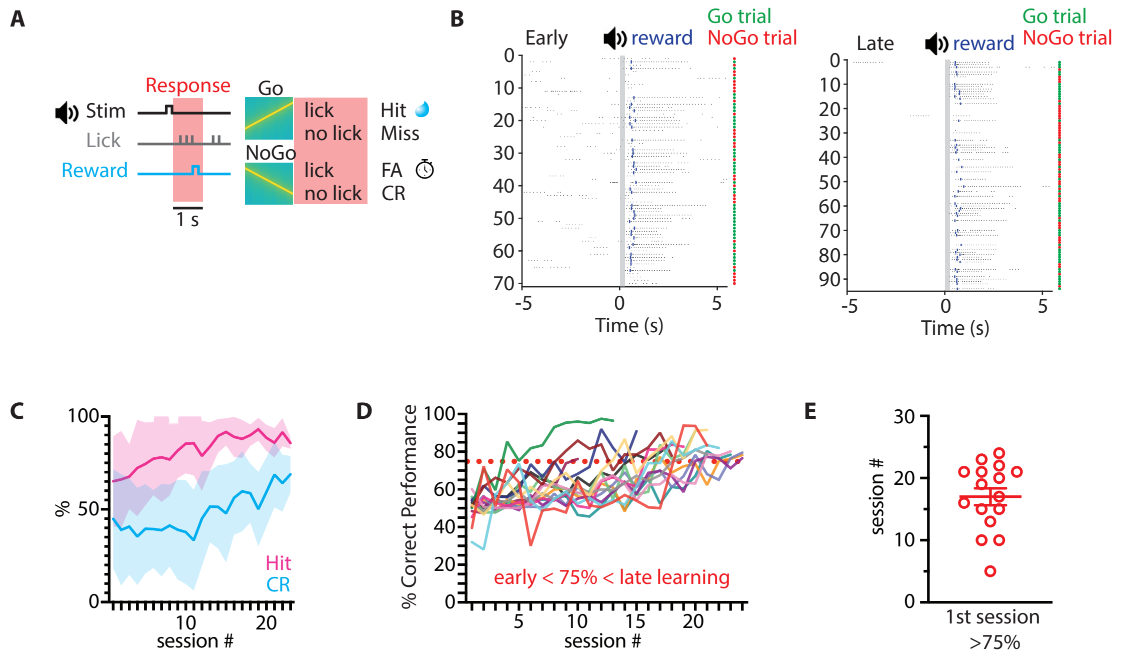

Fig. S2. Auditory discrimination and learning of frequency modulated sweeps. (A) Schematic of the experimental design. Mice learned to discriminate 'Up-sweeps' (8-18 kHz) and 'Down-sweeps' (18-8 kHz). Licking within 1 second of presentation of an Up-sweep resulted in delivery of a sugar water reward while Down-sweeps required withholding licking to avoid a timeout punishment. **(B)** Raster plot of the licking response in an example mouse during early and late learning. Licking behavior became more selective in response to Go stimuli with learning. Shaded grey box, stimulus delivery. Blue line, onset of water valve delivery. Green dot, Go trials. Red dot, NoGo trials. **(C)** Average percentance of Hit and CR trials during learning in the auditory discrimination task. **(D)** Individual learning curves of mice in the auditory discrimination task (n = 16 mice). Correct performance increased from early to late learning until stablizing above 75% (expert threshold). **(E)** Number of sessions to reach performance above 75%.

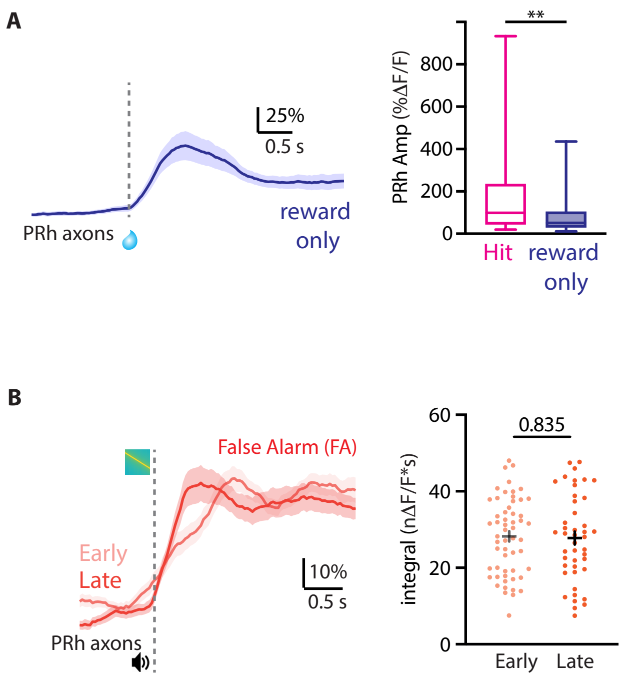

Fig. S3. PRh axonal activity during reward delivery and false alarm trials. (A) (Left) Average PRh axon calcium response during reward delivery alone. In this subset of experiments, sugar water reward was delivered randomly without auditory cue. (Right) Amplitude of calcium responses in PRh axons during Hit trials and reward delivery alone. Mann Whitney test, (**) $P < 0.005$. **(B)** (Left) Average PRh axon calcium response during False Alarm (FA) trials recorded in early (light red) and late (dark red) learning. Grey dotted line, onset of auditory stimulus. (Right) Amplitude of calcium responses in PRh axons in FA trials during early and late learning. Mann Whitney test. Bars represent mean and SEM.

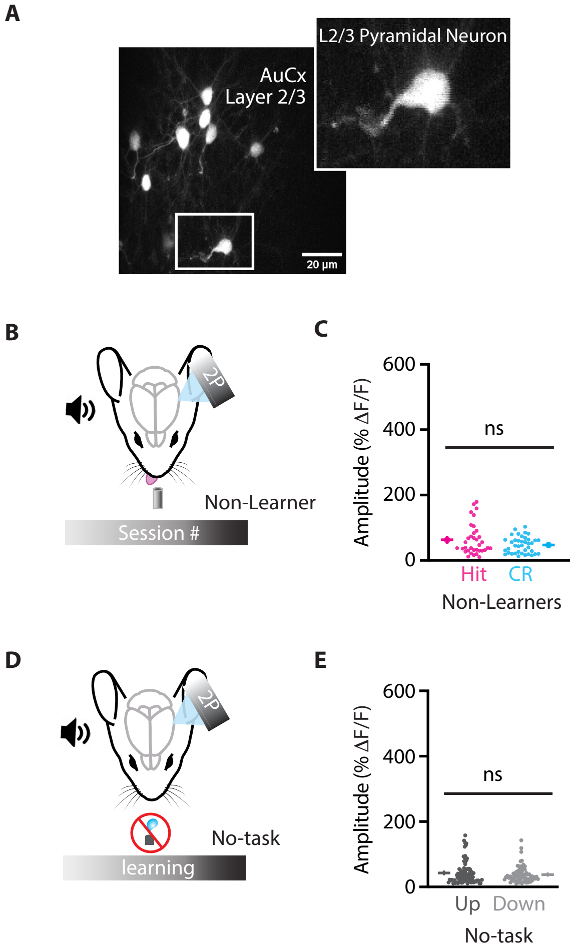

Fig. S4. Signalling in tuft dendrites of L2/3 pyramidal neurons in auditory cortex is modulated by learning and context. (A) Average z-projection acquired in layer 2/3 of the auditory cortex showing expression of GCaMP7f in L2/3 pyramidal neurons. Inset, zoom of L2/3 pyramidal neuron from boxed region. **(B)** Schematic of experimental design. Calcium activity in dendrites in the auditory cortex were recorded in 'non-learner' mice which did not achieve 75% correct performance within 4 weeks. **(C)** Peak amplitude of the calcium response during Hit (magenta) and CR (cyan) trials for 'non-learner' mice (Mann-Whitney test). **(D)** Schematic of experimental design. Calcium activity in dendrites in the auditory cortex were recorded in the 'no-task' condition where the lick port had been removed preventing trained mice to respond. **(E)** Peak amplitude of the calcium response during Up-sweep (dark grey) and Down-sweep (light grey) in the no-task condition after learning (Mann-Whitney test).

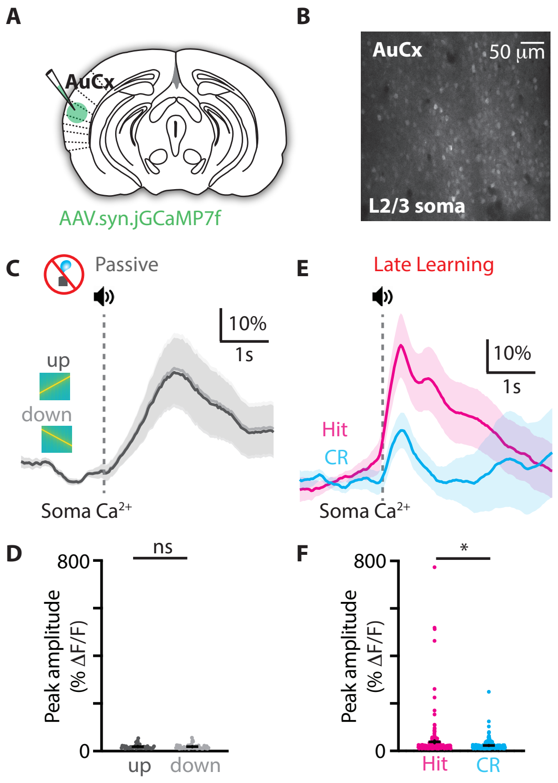

Fig. S5. Somatic activity of L2/3 pyramidal neurons in the auditory cortex during auditory discrimination learning. (A) Schematic of experimental design. The genetically encoded calcium indicator GCaMP7f was injected in layer 2/3 of the auditory cortex. **(B)** Image of somatic calcium imaging plane in layer 2/3 of the auditory cortex (150-250 µm from pia). **(C)** Average calcium response in an example soma during Up-sweeps (dark grey) and Down-sweeps (light grey) in passive listening (prior to learning). Dotted line, onset of the auditory stimulus. **(D)** Peak amplitude of the somatic calcium responses during passive listening. Mann-Whitney test. **(E)** Average calcium response in an example soma during Up-sweeps (dark grey) and Down-sweeps (light grey) in Hit (magenta) and CR (cyan) trials during late leaning. Dotted line, onset of the auditory stimulus. **(F)** Peak amplitude of the somatic calcium responses during Hit (magenta) and CR (cyan) trials in late learning. (*) $P < 0.05$, Mann-Whitney test.

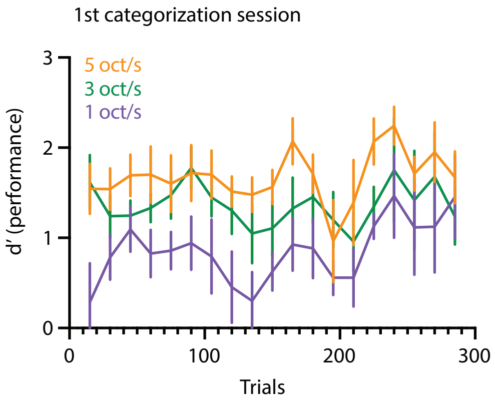

Fig. S6. Behavioural performance in the first categorization session. Discrimination performance during the first categorization session in response to 5 oct/s, 3 oct/s and 1 oct/s stimuli (n = 13 mice; sliding window = 15 trials).
